# Incidence and survival of adenocarcinoma with mixed subtypes in patients with colorectal cancer

**DOI:** 10.1007/s00384-023-04508-4

**Published:** 2023-08-16

**Authors:** Fan Zhang, Boqi Xu, Yao Peng, Zhongqi Mao, Shan Tong

**Affiliations:** Department of General Surgery, The First Affiliated Hospital of Soochow University, Suzhou, China

**Keywords:** Colorectal cancer, Adenocarcinoma with mixed subtypes, Incidence, Survival, Prognostic factor

## Abstract

**Background:**

Limited attention was paid to adenocarcinoma with mixed subtypes (AM) of the colon and rectum due to its low incidence. This study aims to assess the frequency and survival rates of tumors in the population.

**Methods:**

The data were extracted from the Surveillance, Epidemiology, and End Results (SEER) database between 2000 and 2019. The incidence of tumors was evaluated based on patient gender, age, race, and location. Univariate and multivariate Cox analyses were performed to identify risk factors associated with tumor survival. Additionally, a nomogram was constructed using these risk factors to predict cancer-specific survival (CSS) at 1, 2, and 3 years. Receiver operating characteristic (ROC) and calibration curves were applied to examine the model’s accuracy.

**Results:**

The overall incidence of colorectal AM reached its highest level in 2016 (2.350 (95% CI: 2.241–2.462)). AM is more frequent in elderly patients and predominantly located in the rectum. By forest plot for multivariable Cox regression analysis, patient age, the number of regional positive lymph nodes and lymph nodes removed, tumor N/M stage, and postoperative chemotherapy were identified as independent risk indicators for CSS. Nomogram was constructed and validated as a feasible prediction model of CSS in patients with colorectal AM.

**Conclusion:**

The presence of colorectal AM in elderly patients, particularly in the rectum, is frequent and often associated with poor prognosis. Our nomograms can offer a relatively accurate prediction of CSS of patients with AM after tumor resection.

According to the latest cancer statistics, colorectal cancer ranked as the second most commonly diagnosed cancer in women and the third most frequently diagnosed cancer in men [1]. In 2023, approximately 52,550 patients died from colorectal cancer based on incidence from population-based cancer registries and mortality data from the National Center for Health Statistics [2]. Colorectal cancer has become the fourth leading cause of cancer-related death worldwide [1]. Colorectal cancer can be divided into adenocarcinoma, squamous cell carcinoma, and other types according to pathological classification. Adenocarcinoma accounts for 90% of all colorectal cancer cases, originating from the epithelial cells of the colorectal mucosa [3]. Classical adenocarcinoma (CA), mucinous adenocarcinoma (MAC), and signet-ring cell carcinoma (SRCC) are the three main types of adenocarcinoma [4].

Adenocarcinoma with mixed subtypes (AM) is an uncommon form of adenocarcinoma characterized by a mixture of metaplastic and conventional adenocarcinoma components [5]. Morphologically, signet-ring cellular components can be observed within AM [6, 7]. A population-based study has shown that AM was a particularly aggressive histologic subtype of colorectal cancer and had a comparable prognosis to SRCC [8]. However, due to its rarity compared to other pathological subtypes of adenocarcinoma, data on its incidence and survival rates are limited, with few studies focusing on this area. Hence, it is imperative to systematically evaluate colorectal AM incidence and survival in large populations.

The present study aimed to investigate and analyze the incidence and survival of colorectal AM based on data extracted from the Surveillance, Epidemiology, and End Results (SEER) database. The incidence of AM was analyzed based on the patient’s age, sex, race, and tumor site. In addition, a predictive nomogram was constructed to predict the survival rate of patients with colorectal AM.

## Materials and methods

### Data source and patient

A population-based retrospective cohort study was conducted based on Incidence-SEER Research Plus Data, 17 Registries, Nov 2021 Sub (2000–2019). Patients who met the following criteria are included: (1) Adenocarcinoma (CA, SRCC, AM, and MAC) as diagnosed by pathology. (2) Tumors in the colorectum. Patients who met the following criteria are excluded: (1) Patient age, race, and survival are unavailable. (2) Tumor size and TNM stage are missing. (3) The number of regional lymph nodes, positive lymph nodes, and lymph nodes removed is not unknown. (4) Colorectal adenocarcinoma has not been confirmed by positive pathology.

### Search strategy

The present analysis included all patients diagnosed with colorectal cancer and assigned the primary site C18.2–C18.9, C19.9, and C20.9. The topography and histology of the cancer were coded using ICD-O-3 Hist/behave in cancer registries. Our analysis focused on four histologic subtypes — CA (8140/3), AM (8255/3), SRCC (8490/3), and MAC (8480/3). The following variables were extracted from the seer database: patient age, gender, race, tumor site, size, TNM stage (AJCC 6th), chemotherapy, and the number of regional lymph nodes, positive lymph nodes, and lymph nodes removed. Finally, patients with missing or unavailable variables were excluded from the analysis.

### Statistical analysis

All data analyses were performed in R software version 4.4.2 (Institute for Statistics and Mathematics, Vienna, Austria; https://www.r-project.org/). We calculated the incidences in patients with colorectal AM between 2000 and 2019. Then, the colorectal adenocarcinoma was separated into four groups based on histology (CA, SRCC, AM, and MAC) to compare the differences in cancer overall survival (OS) and cancer-specific survival (CSS) based on the Kaplan-Meier curves and log-rank test. Univariate and multivariate Cox regression analyses were performed to identify independent prognostic factors in OS and CSS of colorectal adenocarcinoma.

Patients with colorectal AM were extracted from the above dataset containing AM, SRCC, CA, and MAC. The predictive research was explicitly focused on CSS in colorectal AM patients, who were divided into training and validation groups in a 7:3 ratio. A forest plot was used for multivariable Cox regression analysis of patients with colorectal AM to identify independent risk factors for CSS (*P* < 0.05). A nomogram was constructed based on the independent risk factors to predict the 1-, 2-, and 3-year CSS rates. Calibration curves and receiver operating characteristic (ROC) curves were performed to analyze the feasibility of the nomogram for predicting CSS at 1, 2, and 3 years.

## Results

### Clinicopathological characteristics

**Fig. 1:**
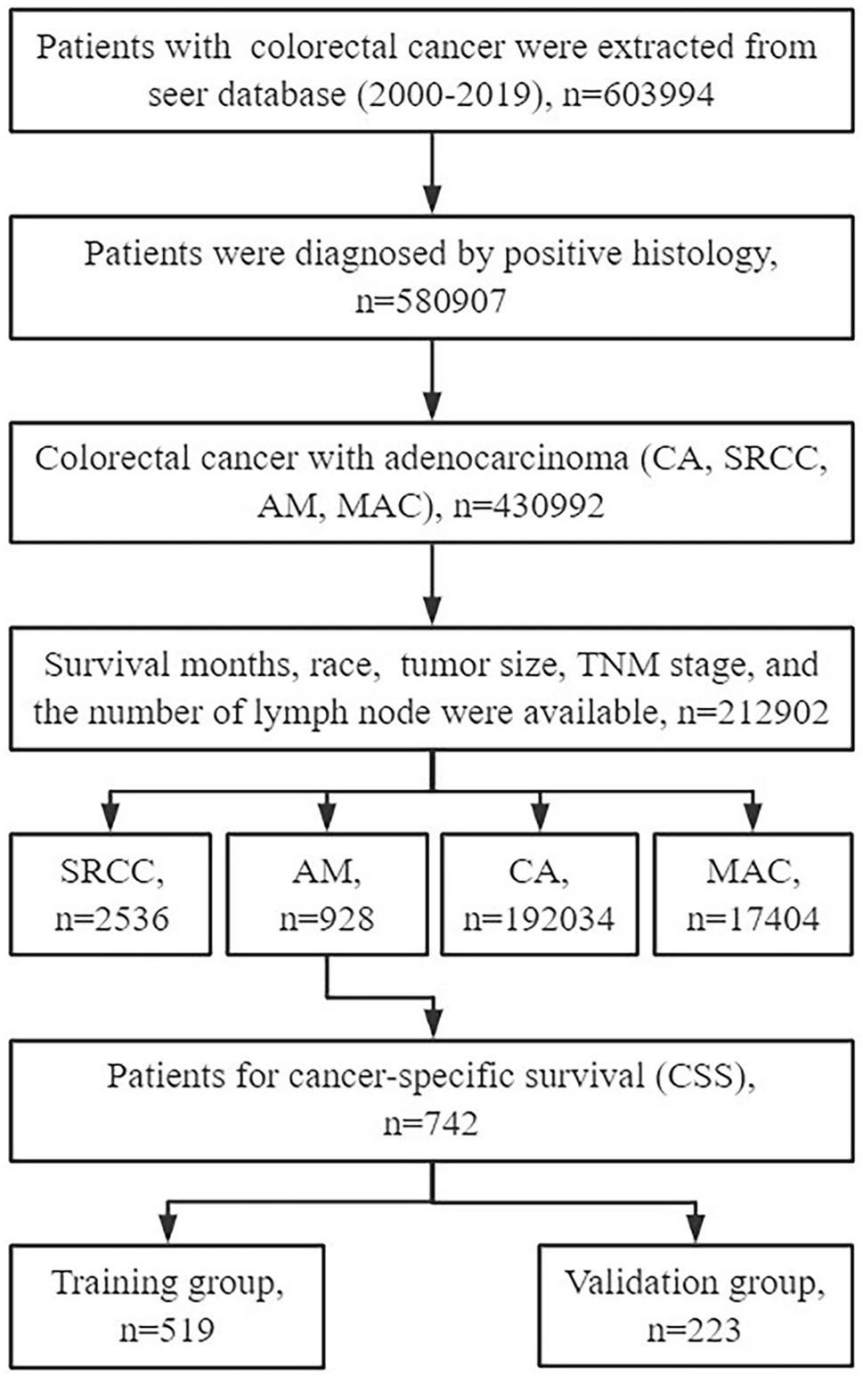
Flow chart depicting the patient selection process. *AM* adenocarcinoma with mixed subtypes, *CA* classical adenocarcinoma, *SRCC* signet-ring cell carcinoma, *MAC* mucinous adenocarcinoma

Results showed that out of the 212,902 patients identified from the SEER database during 2000–2019, 928 had AM, 2536 had SRCC, 192,034 had CA, and 17,404 had MAC. The flow chart of data selection is shown in Fig. 1. The patient’s characteristics are shown in Table 1. The incidence of typical adenocarcinoma is the highest among all types of adenocarcinomas, accounting for 90.2% of cases, which surpasses that of other adenocarcinoma types by a significant margin. Among the four types of adenocarcinomas, AM has the lowest incidence at 0.4%, followed by SRCC at 1.2%. As for the comparison of clinicopathological variables, significant differences were observed between groups (*P* < 0.05).

**Table 1:** Comparisons of the clinicopathologic features between CA, AM, SRCC, and MAC

	**CA**	**AM**	**SRCC**	**MAC**	**Overall**	***P***
***N***	192,034 (90.2)	928 (0.4)	2536 (1.2)	17,404 (8.2)	212,902 (1.0)	
**Age**						<0.05
<60	58,836 (30.6)	296 (31.9)	912 (36.0)	4822 (27.7)	64,866 (30.5)	
≥60	133,198 (69.4)	632 (68.1)	1624 (64.0)	12,582 (72.3)	148,036 (69.5)	
**Race**						<0.05
Black	20,309 (10.6)	76 (8.2)	220 (8.7)	1688 (9.7)	22,293 (10.5)	
White	153,037 (79.7)	759 (81.8)	2088 (82.3)	14,492 (83.3)	170,376 (80.0)	
Other	18,688 (9.7)	93 (10.0)	228 (9.0)	1224 (7.0)	20,233 (9.5)	
**Size**						<0.05
<40	70,283 (36.6)	213 (23.0)	514 (20.3)	4540 (26.1)	75,550 (35.5)	
40–80	88,608 (46.1)	484 (52.2)	1261 (49.7)	9018 (51.8)	99,371 (46.7)	
>80	33,143 (17.3)	231 (24.9)	761 (30.0)	3846 (22.1)	37,981 (17.8)	
**Site**						<0.05
Colon	127,214 (66.2)	674 (72.6)	1832 (72.2)	13,573 (78.0)	143,293 (67.3)	
Rectum	64,820 (33.8)	254 (27.4)	704 (27.8)	3831 (22.0)	69,609 (32.7)	
**Nodes removed**						<0.05
0	24,487 (12.8)	93 (10.0)	450 (17.7)	1229 (7.1)	26,259 (12.3)	
1–3	6688 (3.5)	23 (2.5)	76 (3.0)	568 (3.3)	7355 (3.5)	
≥4	160,859 (83.8)	812 (87.5)	2010 (79.3)	15,607 (89.7)	179,288 (84.2)	
**Positive nodes**						<0.05
<20	166,693 (86.8)	808 (87.1)	1955 (77.1)	16,034 (92.1)	185,490 (87.1)	
20–40	985 (0.5)	27 (2.9)	128 (5.0)	195 (1.1)	1335 (0.6)	
>40	24,356 (12.7)	93 (10.0)	453 (17.9)	1175 (6.8)	26,077 (12.2)	
**Regional nodes**						<0.05
<30	174,986 (91.1)	802 (86.4)	2223 (87.7)	15,416 (88.6)	193,427 (90.9)	
30–60	15,092 (7.9)	107 (11.5)	268 (10.6)	1754 (10.1)	17,221 (8.1)	
>60	1956 (1.0)	19 (2.0)	45 (1.8)	234 (1.3)	2254 (1.1)	
**Sex**						<0.05
Female	88,764 (46.2)	402 (43.3)	1174 (46.3)	8560 (49.2)	98,900 (46.5)	
Male	103,270 (53.8)	526 (56.7)	1362 (53.7)	8844 (50.8)	114,002 (53.5)	
**M stage**						<0.05
M0	159,343 (83.0)	681 (73.4)	1814 (71.5)	14,593 (83.8)	176,431 (82.9)	
M1	32,691 (17.0)	247 (26.6)	722 (28.5)	2811 (16.2)	36,471 (17.1)	
**N stage**						<0.05
N0	108,206 (56.3)	292 (31.5)	839 (33.1)	9305 (53.5)	118,642 (55.7)	
N1	53,669 (27.9)	229 (24.7)	594 (23.4)	4535 (26.1)	59,027 (27.7)	
N2	30,159 (15.7)	407 (43.9)	1103 (43.5)	3564 (20.5)	35,233 (16.5)	
**T stage**						<0.05
T0	296 (0.2)	3 (0.3)	3 (0.1)	26 (0.1)	328 (0.2)	
T1	21,086 (11.0)	45 (4.8)	210 (8.3)	1057 (6.1)	22,398 (10.5)	
T2	27,646 (14.4)	60 (6.5)	123 (4.9)	1934 (11.1)	29,763 (14.0)	
T3	114,283 (59.5)	498 (53.7)	1275 (50.3)	10,558 (60.7)	126,614 (59.5)	
T4	28,723 (15.0)	322 (34.7)	925 (36.5)	3829 (22.0)	33,799 (15.9)	
**Chemotherapy**						<0.05
No/unknown	107,346 (55.9)	441 (47.5)	1171 (46.2)	10,073 (57.9)	119,031 (55.9)	
Yes	84,688 (44.1)	487 (52.5)	1365 (53.8)	7331 (42.1)	93,871 (44.1)	

*AM* adenocarcinoma with mixed subtypes, *CA* classical adenocarcinoma, *SRCC* signet-ring cell carcinoma, *MAC* mucinous adenocarcinoma

### Incidence and trend of AM

**Fig. 2:**
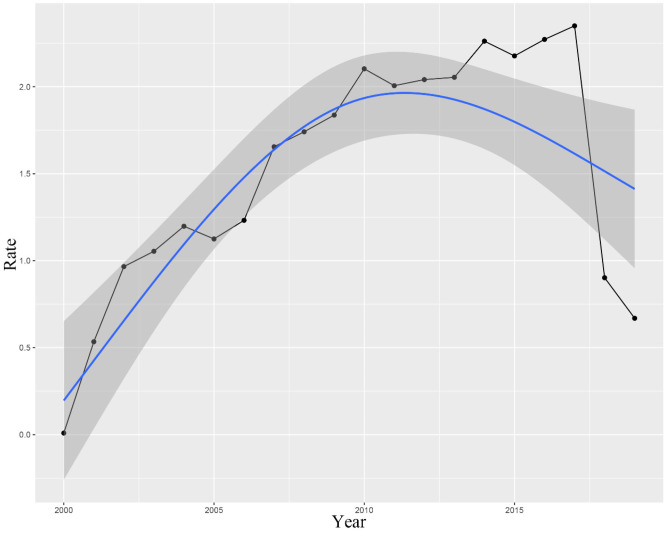
Incidence rates and trend of colorectal adenocarcinoma with mixed subtypes in SEER 17 registries (per 1,000,000 person-years)

**Fig. 3:**
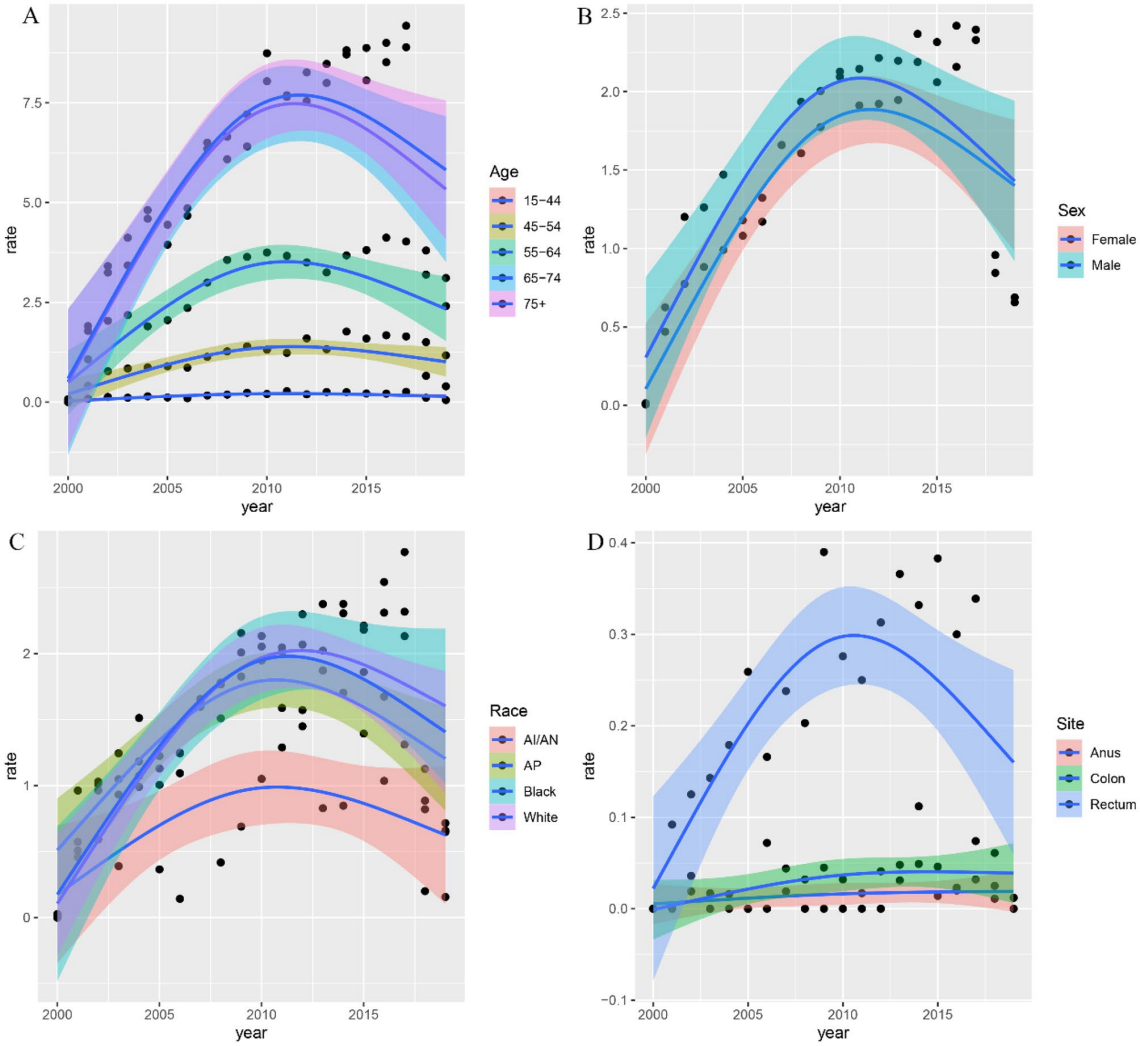
Incidence and trends of colorectal adenocarcinoma with mixed subtypes by subgroups: **A** age, **B** sex, **C** race, and **D** site

The incidence and trends of colorectal AM and its differences among patient subgroups were assessed in Figs. 2 and 3. The overall incidence and movement of colorectal AM had been increasing before 2017 and subsequently decreased. The highest overall incidence in the colorectum was 2.350 (95% CI: 2.241–2.462) per 100,000 person-years. Figure 3 shows the differences in tumor incidence among patients with different ages, gender, race, and tumor site. Among the older population, the incidence was higher than the young population (Fig. 3A). No essential differences in the overall incidence and trend of tumors were observed between male and female cases (Fig. 3B). The incidence was 3.682 (95% CI: 0.191–7.293) for the white population and 4.706 (95% CI: 0.703–8.868) for the black population per 100,000 person-years. Blacks had the highest incidence in the overall population (Fig. 3C). As for the site of the tumor occurrence, AM mainly occurred in the rectum. No apparent differences were observed in the incidence and trends of AM between the colon and anus (Fig. 3D).

### Survival analysis between different pathological subgroups

**Fig. 4:**
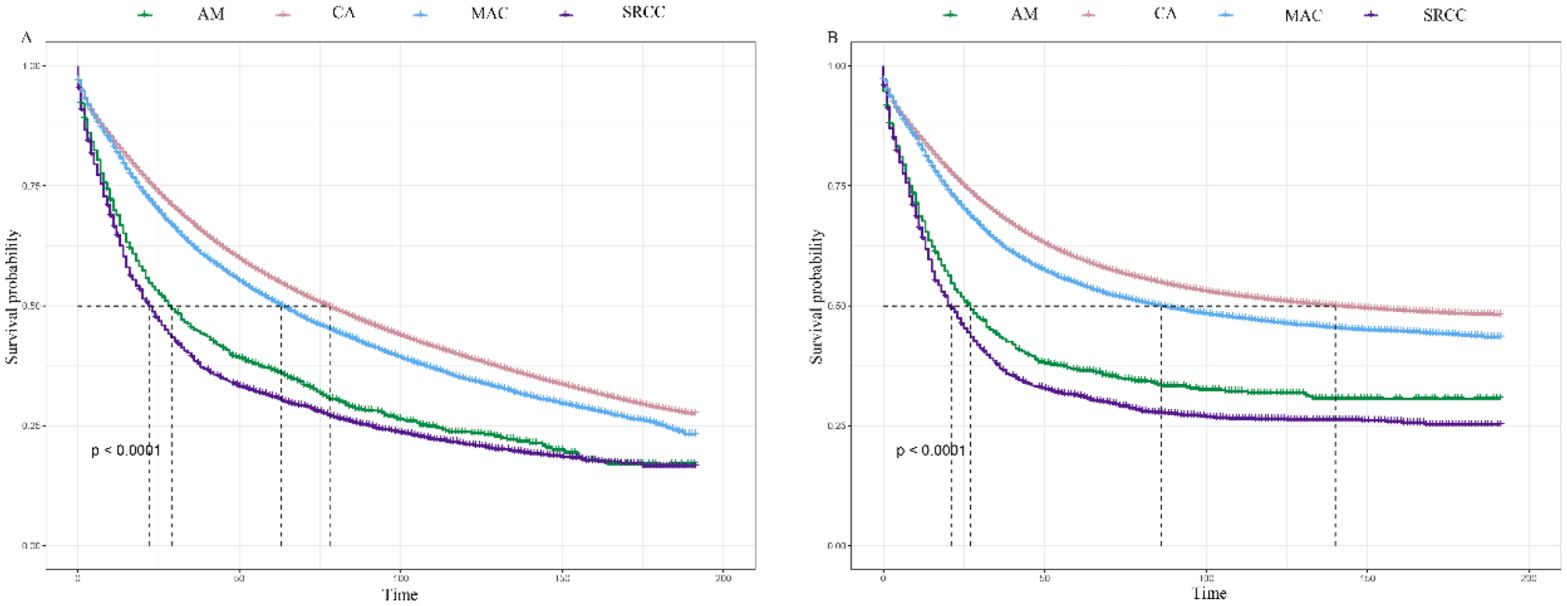
Kaplan-Meier (KM) survival curves comparing the overall survival (**A**) and cancer-specific survival (CSS) (**B**) of patients in different pathological subgroups. AM: adenocarcinoma with mixed subtypes; CA: classical adenocarcinoma; SRCC: signet-ring cell carcinoma; MAC: mucinous adenocarcinoma

The present study employed Kaplan-Meier curves to analyze the relationship between tumor pathology and clinical outcomes such as CSS and OS. Notably, the CSS and OS of patients with CA and MAC are significantly higher than those of patients with SRCC and AM, while patients with AM and SRCC displayed similar prognoses (Fig. 4). The 1-, 2-, and 3-year OS and CSS are shown in Tables 2 and 3. In the entire population, the median OS in CA, AM, SRCC, and MAC patients were 78 months (77–78 months), 29 months (25–33 months), 22 months (20–24 months), and 63 months (61–65 months), respectively. In the population containing patients who died from colorectal cancer, the median CSS in CA, AM, SRCC, and MAC patients were 140 months (135–145 months), 27 months (22–32 months), 21 months (19–23 months), and 86 (80–93 months), respectively. Lastly, univariate and multivariate Cox analyses were performed to identify independent prognostic factors of OS and CSS in patients with colorectal CA, AM, SRCC, and MAC (Table 4). Patients with CA and MAC benefited more in terms of OS and CSS compared with those with SRCC and AM.

**Table 2:** The 1-, 2-, and 3-year overall survival (OS) rates of patients with CA, AM, SRCC, and MAC

**Pathological characteristics**	**1-year OS rate**	**2-year OS rate**	**3-year OS rate**
CA	0.835 (0.834–0.837)	0.743 (0.741–0.745)	0.668 (0.666–0.670)
AM	0.689 (0.660–0.719)	0.533 (0.502–0.566)	0.450 (0.419–0.483)
MAC	0.820 (0.814–0.825)	0.705 (0.699–0.712)	0.385 (0.367–0.405)
SRCC	0.645 (0.627–0.664)	0.475 (0.456–0.495)	0.385 (0.367–0.405)

*AM* adenocarcinoma with mixed subtypes, *CA* classical adenocarcinoma, *SRCC* signet-ring cell carcinoma, *MAC* mucinous adenocarcinoma, *OS* overall survival

**Table 3:** The 1-, 2-, and 3-year cancer-specific survival (CSS) rates of patients with CA, AM, SRCC, and MAC

**Pathological characteristics**	**1-year CSS rate**	**2-year CSS rate**	**3-year CSS rate**
CA	0.845 (0.843–0.847)	0.756 (0.754–0.758)	0.688 (0.686–0.690)
AM	0.677 (0.644–0.712)	0.519 (0.485–0.557)	0.435 (0.400–0.472)
MAC	0.824 (0.818–0.831)	0.709 (0.701–0.717)	0.629 (0.621–0.637)
SRCC	0.640 (0.620–0.661)	0.461 (0.440–0.483)	0.370 (0.350–0.391)

*AM* adenocarcinoma with mixed subtypes, *CA* classical adenocarcinoma, *SRCC* signet-ring cell carcinoma, *MAC* mucinous adenocarcinoma, *CSS* cancer-specific survival

**Table 4:** Univariate and multivariate Cox hazard proportional model analysis of patients with adenocarcinoma (AM, CA, SRCC, and MAC)

	**Univariate analysis**						**Multivariate analysis**						
	**OS**			**CSS**			**OS**				**CSS**		
	HR	95% CI	*P*	HR	95% CI	*P*	HR		95% CI	*P*	HR	95% CI	*P*
**Age**													
<60	Reference			Reference			Reference				Reference		
≥60	1.93	1.90–1.95	<0.001	1.63	1.61–1.65	<0.001	2.05		2.03–2.07	<0.001	1.29	1.75–1.80	<0.001
**Race**													
White	Reference			Reference			Reference				Reference		
Black	1.11	1.09–1.12	<0.001	1.18	1.16–1.20	<0.001	1.10		1.08–1.12	<0.001	1.03	1.12–1.16	0.007
Other	0.81	0.80–0.83	<0.001	0.83	0.82–0.85	<0.001	0.82		0.81–0.84	<0.001	0.95	0.83–0.86	<0.001
**Size**													
<40	Reference			Reference			Reference				Reference		
40–80	1.29	1.27–1.30	<0.001	1.49	1.47–1.52	<0.001	1.11		1.10–1.12	<0.001	1.09	1.13–1.17	<0.001
>80	1.62	1.62–1.67	<0.001	2.07	2.03–2.10	<0.001	1.20		1.18–1.21	<0.001	1.22	1.27–1.31	<0.001
**Site**													
Colon	Reference			Reference			Reference				Reference		
Rectum	0.91	0.90–0.92	<0.001	0.97	0.96–0.99	<0.001	0.98		0.97–0.99	0.002	0.91	0.99–1.01	0.928
**Nodes removed**													
0	Reference			Reference			Reference				Reference		
1–3	0.55	0.53–0.56	<0.001	0.49	0.48–0.51	<0.001	0.90		0.87–0.94	<0.001	0.79	0.94–1.03	0.549
≥4	0.43	0.42–0.44	<0.001	0.36	0.36–0.37	<0.001	0.64		0.62–0.66	<0.001	0.68	0.59–0.64	<0.001
**Positive nodes**													
<20	Reference			Reference			Reference				Reference		
20–40	3.44	3.28–3.61	<0.001	4.10	3.90–4.31	<0.001	1.77	1.69–1.86		<0.001	1.44	1.65–1.83	<0.001
>40	2.32	2.29–2.35	<0.001	2.78	2.74–2.83	<0.001	1.62	1.57–1.67		<0.001	1.36	1.69–1.82	<0.001
**Regional nodes**													
<30	Reference			Reference			Reference				Reference		
30–60	0.71	0.70–0.72	<0.001	0.65	0.64–0.67	<0.001	0.75	0.74–0.77		<0.001	0.90	0.68–0.72	<0.001
>60	1.12	1.07–1.17	<0.001	1.21	1.15–1.28	<0.001	0.88	0.84–0.92		<0.001	0.95	0.83–0.92	<0.001
**Sex**													
Female	Reference			Reference			Reference				Reference		
Male	1.07	1.06–1.08	<0.001	1.06	1.05–1.08	<0.001	1.07	1.06–1.08		<0.001	0.96	1.03–1.05	<0.001
**M stage**													
M0	Reference			Reference			Reference				Reference		
M1	3.93	3.88–3.97	<0.001	5.28	5.21–5.35	<0.001	3.40	3.35–3.44		<0.001	1.79	3.72–3.83	<0.001
**N stage**													
N0	Reference			Reference			Reference				Reference		
N1	1.25	1.24–1.26	<0.001	1.60	1.58–1.62	<0.001	1.42	1.41–1.44		<0.001	1.26	1.62–1.67	<0.001
N2	1.91	1.89–1.94	<0.001	2.68	2.64–2.72	<0.001	2.01	1.98–2.04		<0.001	1.54	2.46–2.55	<0.001
**T stage**													
T0	Reference			Reference			Reference				Reference		
T1	0.22	0.20–0.25	<0.001	0.18	0.17–0.20	<0.001	0.56	0.51–0.62		<0.001	0.58	0.43–0.54	<0.001
T2	0.15	0.14–0.16	<0.001	0.08	0.07–0.09	<0.001	0.55	0.49–0.60		<0.001	0.47	0.32–0.39	<0.001
T3	0.22	0.20–0.25	<0.001	0.19	0.17–0.21	<0.001	0.75	0.68–0.83		<0.001	0.60	0.59–0.73	<0.001
T4	0.44	0.40–0.49	<0.001	0.45	0.41–0.50	<0.001	1.13	1.02–1.24		0.04	0.79	0.94–1.16	0.51
**CHE-**													
No/Un	Reference			Reference			Reference				Reference		
Yes	0.83	0.83–0.84	<0.001	1.04	1.03–1.05	<0.001	0.53	0.52–0.54		<0.001	0.56	0.54–0.55	<0.001
**Subtype**													
AM	Reference			Reference			Reference				Reference		
CA	0.58	0.55–0.62	<0.001	0.62	0.48–0.55	<0.001	0.74	0.69–0.79		<0.001	0.69	0.67–0.79	<0.001
SRCC	1.12	1.04–1.20	0.01	1.02	0.55–0.64	0.004	1.07	1.00–1.15		0.120	1.02	1.01–1.20	0.062
MAC	0.66	0.62–0.70	<0.001	0.66	1.07–1.27	<0.001	0.79	0.74–0.85		<0.001	0.72	0.74–0.86	<0.001

*AM* adenocarcinoma with mixed subtypes, *CA* classical adenocarcinoma, *SRCC* signet-ring cell carcinoma, *MAC* mucinous adenocarcinoma, *OS* overall survival, *CSS* cancer-specific survival, *CHE-*: chemotherapy, *No/Un* no/unknown

### Construction of the nomogram for predicting CSS of colorectal AM

**Fig. 5:**
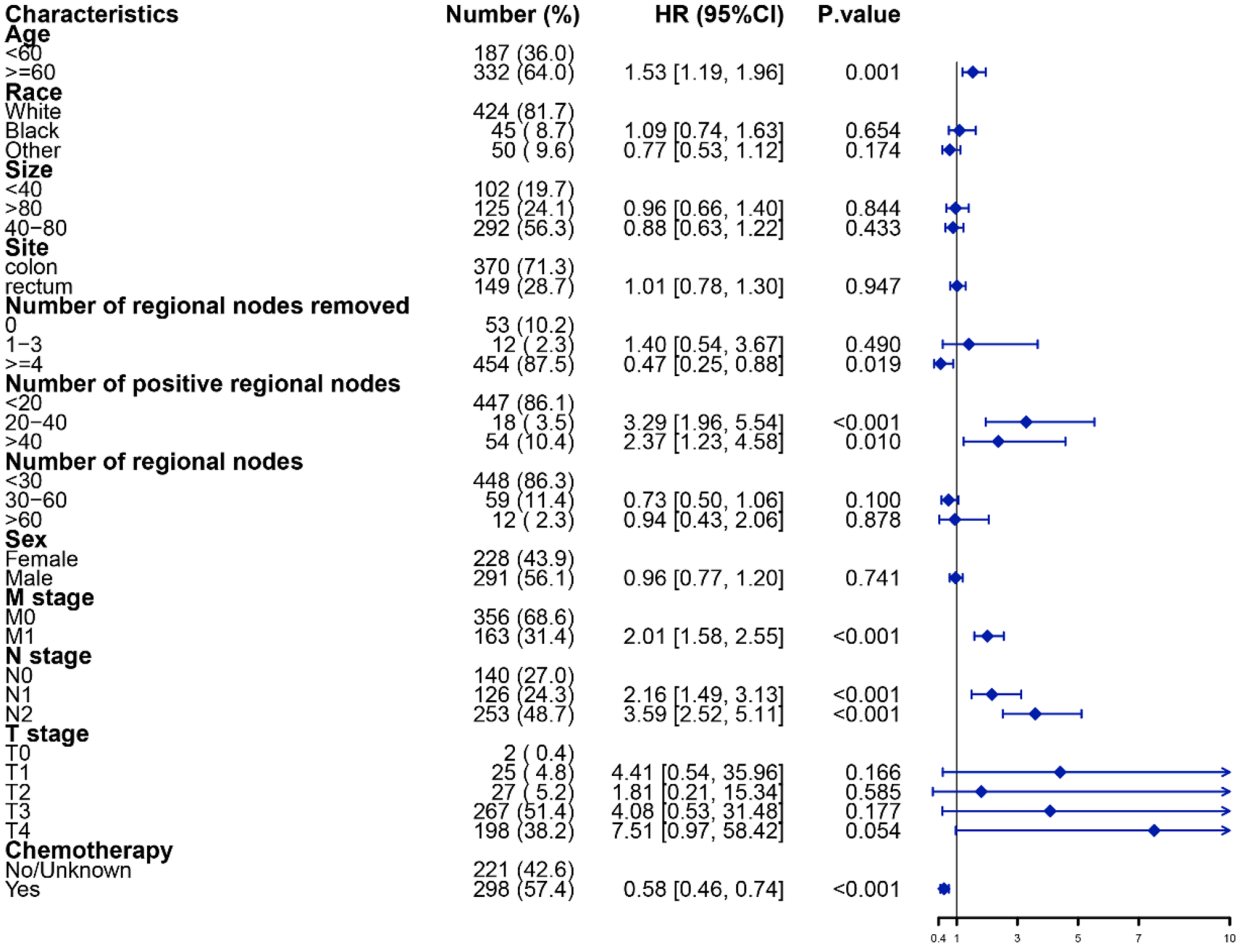
Forest plot for multivariable Cox regression analysis of CSS in patients with colorectal adenocarcinoma with mixed subtypes (training group)

**Fig. 6:**
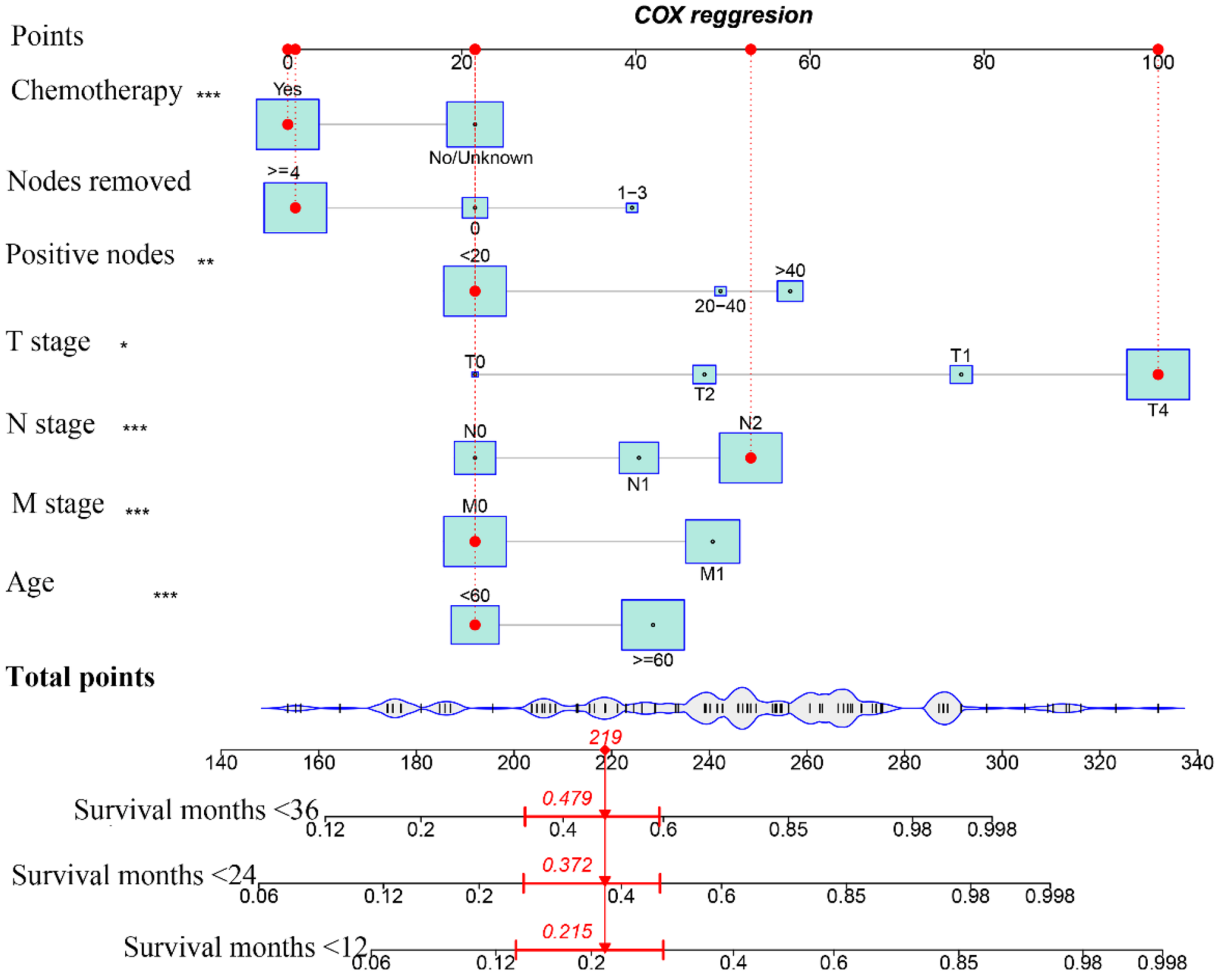
A prognostic nomogram predicting the cancer-specific survival (CSS) of patients with colorectal adenocarcinoma with mixed subtypes for the 12, 24, and 36 months (training group)

**Fig. 7:**
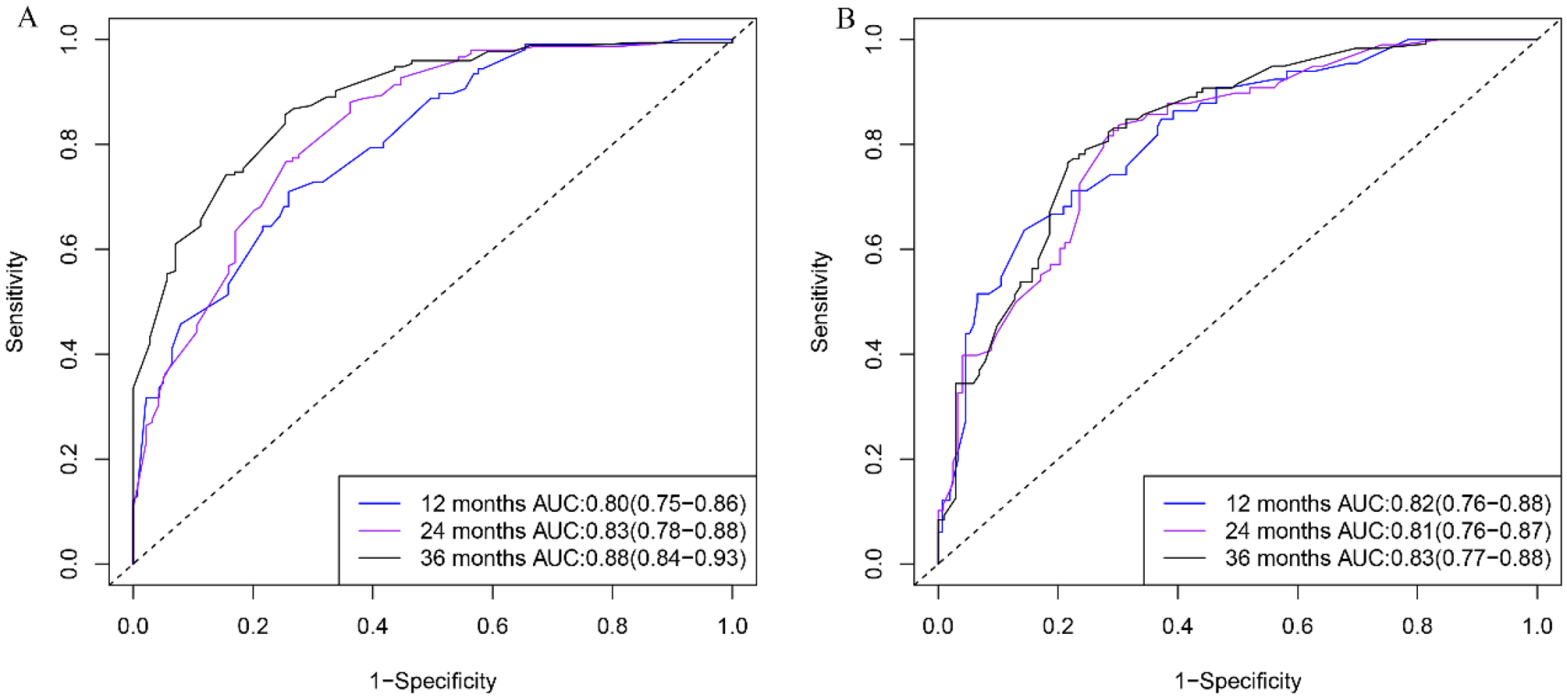
Time-dependent receiver operating characteristic (ROC) curves of cancer-specific survival (CSS) for the 12, 24, and 36 months in the training (**A**) and validation (**B**) groups in colorectal adenocarcinoma with mixed subtypes. AUC: area under the curve

**Fig. 8:**
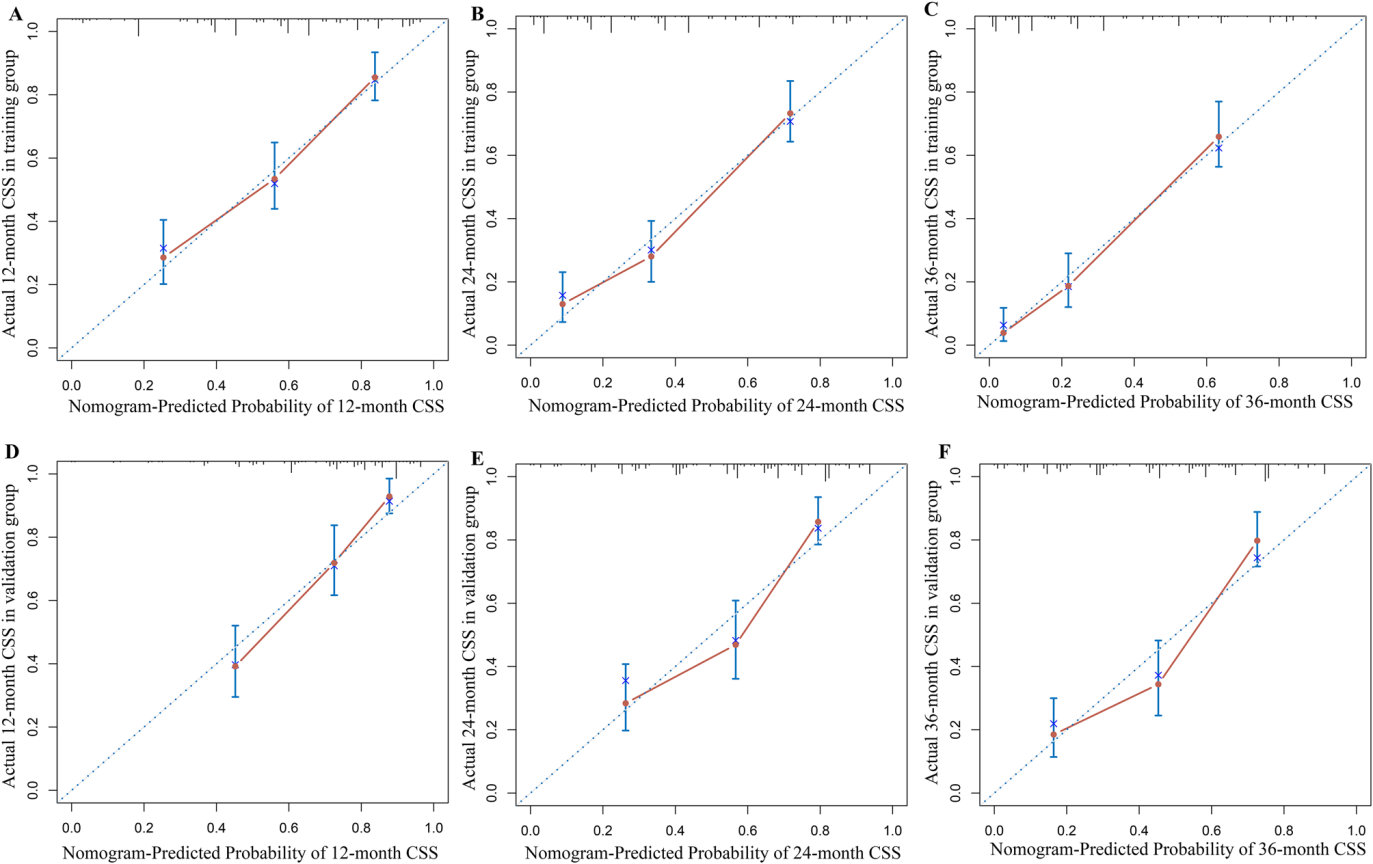
Calibration plots of 12-, 24-, and 36-month cancer-specific survival (CSS) for adenocarcinoma with mixed subtypes in the training (**A**–**C**) and validation (**D**–**F**) groups

Patient age, tumor TNM stage, postoperative chemotherapy, and the number of regional positive lymph nodes and lymph nodes removed were identified as risk factors for CSS by Cox regression forest plots (Fig. 5). A total of 742 patients with colorectal AM were extracted to analyze and predict the CSS at 1, 2, and 3 years. Of these, 519 patients were assigned to the training group and 223 to the validation group. The specific clinicopathological features of the two groups are shown in Table 5. The Chi-square and Fisher’s exact test indicated no statistically significant difference between the training and validation groups for all variables (*P* > 0.05). Figure 6 shows a novel nomogram based on risk factors confirmed through multivariate Cox regression in the training group to determine the 1-, 2-, and 3-year CSS of patients with AM. In addition, the C-index value of CSS in colorectal AM was shown in Fig. 7 (training group: 1 year = 0.80, 2 years = 0.83, 3 years = 0.88; validation group: 1 year = 0.82, 2 years = 0.813, 3 years = 0.83), which also showed good discrimination in predicting the CSS of colorectal patients with AM. Calibration curves were plotted to reveal the high coherence between the nomogram-predicted and actual CSS at 1, 2, and 3 years (Fig. 8).

**Table 5:** Baseline clinical characteristics of patients diagnosed as adenocarcinoma with mixed subtypes in training and validation groups

	**Training (*N* = 519)**	**Validation (*N* = 223)**	**Overall (*N* = 742)**	***χ***^**2**^	***P***
**Age**				0.475	0.491
<60	187 (36.0%)	87 (39.0%)	274 (36.9%)		
≥60	332 (64.0%)	136 (61.0%)	468 (63.1%)		
**Race**				1.354	0.508
White	424 (81.7%)	182 (81.6%)	606 (81.7%)		
Black	45 (8.7%)	15 (6.7%)	60 (8.1%)		
Other	50 (9.6%)	26 (11.7%)	76 (10.2%)		
**Size**				4.783	0.091
<40	102 (19.7%)	53 (23.8%)	155 (20.9%)		
40–80	292 (56.3%)	106 (47.5%)	398 (53.6%)		
>80	125 (24.1%)	64 (28.7%)	189 (25.5%)		
**Site**				0.275	0.600
Colon	370 (71.3%)	154 (69.1%)	524 (70.6%)		
Rectum	149 (28.7%)	69 (30.9%)	218 (29.4%)		
**Nodes removed**				0.977	0.614
0	53 (10.2%)	23 (10.3%)	76 (10.2%)		
1–3	12 (2.3%)	8 (3.6%)	20 (2.7%)		
≥4	454 (87.5%)	192 (86.1%)	646 (87.1%)		
**Positive nodes**				0.055	0.973
<20	447 (86.1%)	193 (86.5%)	640 (86.3%)		
20–40	18 (3.5%)	7 (3.1%)	25 (3.4%)		
>40	54 (10.4%)	23 (10.3%)	77 (10.4%)		
**Regional nodes**				0.569	0.752
<30	448 (86.3%)	190 (85.2%)	638 (86.0%)		
30–60	59 (11.4%)	29 (13.0%)	88 (11.9%)		
>60	12 (2.3%)	4 (1.8%)	16 (2.2%)		
**Sex**				0.500	0.480
Female	228 (43.9%)	91 (40.8%)	319 (43.0%)		
Male	291 (56.1%)	132 (59.2%)	423 (57.0%)		
**M stage**				1.594	0.207
M0	356 (68.6%)	164 (73.5%)	520 (70.1%)		
M1	163 (31.4%)	59 (26.5%)	222 (29.9%)		
**N stage**				1.727	0.422
N0	140 (27.0%)	66 (29.6%)	206 (27.8%)		
N1	126 (24.3%)	60 (26.9%)	186 (25.1%)		
N2	253 (48.7%)	97 (43.5%)	350 (47.2%)		
**T stage**				1.724	0.786
T0	2 (0.4%)	1 (0.4%)	3 (0.4%)		
T1	25 (4.8%)	8 (3.6%)	33 (4.4%)		
T2	27 (5.2%)	13 (5.8%)	40 (5.4%)		
T3	267 (51.4%)	124 (55.6%)	391 (52.7%)		
T4	198 (38.2%)	77 (34.5%)	275 (37.1%)		
**Chemotherapy**				0.511	0.475
No/unknown	221 (42.6%)	102 (45.7%)	323 (43.5%)		
Yes	298 (57.4%)	121 (54.3%)	419 (56.5%)

## Discussion

According to current knowledge, there have been limited reports on the occurrence and survival rates of colorectal AM [8], and the clinicopathological features of AM remain unclear. Additionally, there is a lack of unified pathological diagnostic criteria [6, 9]. In view of this, this study aimed to address the lack of knowledge regarding the incidence rate and survival of colorectal AM.

Utilizing data from the SEER database, the study focused on the incidence rate and survival of patients with colorectal AM. An upward trend in the incidence of AM in colorectal cancer was observed between 2000 and 2017. Factors such as patient age, race, and tumor site are associated with cancer incidence, with a higher frequency observed in patients aged over 65 years and with rectal tumors. In addition, our research showed that colorectal AM and SRCC were considered to be related to poor prognosis compared to colorectal CA and MAC. And a nomogram for 1-, 2-, and 3-year CSS prediction was constructed and validated as a reliable model for patients with colorectal AM in our study.

According to our investigation, the older population is more susceptible to developing colorectal AM than their younger population. Previous studies have indicated a noteworthy increase in the frequency of colorectal adenocarcinoma among the elderly population [10–12], but no relevant research has been conducted on the particular difference in the incidence rate of colorectal AM between young and elderly cohorts. With regard to the predilection sites of colorectal AM, the incidence rate of rectal tumors is significantly higher than that of the colon and anus. In 2023, there were an estimated 153,020 new cases of colorectal cancer in the USA, of which 106,970 cases were tumors in the colon and 46,050 were tumors in the rectum, demonstrating a higher likelihood of colorectal tumors occurring in the rectum [2]. Due to the scarcity of literature on the particular pathological type of AM in colorectal cancer, further research is necessary to determine the incidence of AM in different subgroups.

Numerous studies have been conducted to report the clinical and pathological features of gastric AM. However, there is a dearth of the relevant literature concerning colorectal AM [13–15]. Since AM has been known to include signet-ring cellular components [6, 7], patients with colorectal SRCC were extracted from the database and compared with patients with colorectal AM. A population-based study carried out by Benesch M. G. K. et al. demonstrated that SRCC had a worse prognosis than conventional adenocarcinoma [16]. In line with our research, OS and CSS were significantly higher in patients with CA than those with SRCC. Additionally, there is no significant difference in OS between patients with SRCC and AM, as depicted in Fig. 4. Colorectal AM was associated with poor survival.

According to the forest plot depicting the outcomes of multivariate Cox regression analysis for CSS of colorectal AM, patient age, tumor T/N/M stage, and the number of positive regional lymph nodes and lymph nodes removed were identified as independent risk factors. The prognosis of colorectal cancer was positively associated with the removal of more than four regional lymph nodes, whereas there was no significant difference in tumor prognosis between the removal of less than three lymph nodes and no lymph node removal, which was consistent with previous studies [17, 18].

Concerning tumor T/N/M stage, the poor prognosis was easily detected, especially in patients with T4/N2/M1 stage. Bianchi G. et al. conducted a retrospective study from 2002 to 2018, involving 2652 patients with I–III stage colorectal adenocarcinoma. The study revealed that the N stage was significantly associated with lymphovascular invasion [19], which played an essential role in the survival rate of patients with colorectal cancer [19, 20]. As per the sixth edition of AJCC staging for colorectal cancer, the presence of distant metastasis in the M1 stage indicates a poorer tumor prognosis compared to those without distant metastasis [21–23].

The association between tumor N stage and the number of regional lymph nodes has been established, with numerous studies indicating that the number of positive lymph nodes was a crucial risk factor for tumor survival [24, 25]. The present study revealed that a more significant number of regional lymph nodes removed and fewer regional positive lymph nodes resulted in a better prognosis of tumor patients at 1, 2, and 3 years after surgery. A retrospective cohort study consisting of 2198 patients was performed by Hu et al., demonstrating that log odds of positive lymph nodes exhibited a satisfying predictive ability for patient survival, even better than tumor N stage [26]. Consistent with our research, there was a correlation between the number of lymph node resections and postoperative survival of patients [27–29]. Complete mesocolic excision (CME) is currently recommended as one of the surgical methods for treating colon cancer [30, 31], which involves the removal of more lymph nodes than conventional surgery, including mesenteric lymph nodes and central vascular ligation (CVL) [32]. Studies have shown that lower tumor recurrence rates and higher survival rates were found in these patients undergoing CME [33].

Retrospective studies utilizing the SEER database are subject to certain inherent limitations. While patients from the SEER database were categorized into chemotherapy and nonchemotherapy groups, specific details regarding the chemotherapy regimen were unavailable. Additionally, it is impossible to ascertain whether patients included in the study have synchronous tumors in other regions or comorbidities, which inevitably impact the postoperative prognosis of tumor patients. Finally, preoperative neoadjuvant therapy, the level of carcinoembryonic antigen (CEA) and carbohydrate antigen 19-9 (CA19-9), and specific details regarding tumor pathology are known to be associated with tumor prognosis [34–36]. However, none of the relevant data were considered in this study.

## Conclusion

To summarize, AM is a type of colorectal cancer typically with a negative prognosis and is more prevalent among elderly individuals and in the rectum. Patients’ age, chemotherapy, tumor T/N/M stage, and the number of positive lymph nodes and lymph nodes removed are strongly linked to the OS and CSS of patients with colorectal AM. The nomogram established based on these risk factors has been developed and validated for predictive survival in colorectal AM patients. Nevertheless, further research is necessary to gain a better understanding of the clinical and pathological characteristics of this particular type of colorectal cancer.

## Data Availability

Publicly available datasets were analyzed in this study. This data can be found here: Surveillance, Epidemiology, and End Results (SEER) database (https://seer.cancer.gov/).
